# Hypertension among Current Cigarette Smokers Visiting Outpatient Department of a Tertiary Care Centre: A Descriptive Cross-sectional Study

**DOI:** 10.31729/jnma.7424

**Published:** 2022-04-30

**Authors:** Chandra Kala Rai, Rita Kafle, Sarbada Makaju

**Affiliations:** 1 Department of Physiology, Kathmandu Medical College and Teaching Hospital, Duwakot, Bhaktapur, Nepal; 2 Department of General Practice and Emergency, Kathmandu Medical College and Teaching Hospital, Duwakot, Bhaktapur, Nepal; 3 Department of Anatomy, Kathmandu Medical College and Teaching Hospital, Duwakot, Bhaktapur, Nepal

**Keywords:** *habits*, *hypertension*, *prevalence*, *smokers*

## Abstract

**Introduction:**

Hypertension is a common physical condition with high blood pressure for a prolonged period. Long risk factors like age, overweight, high dietary salt intake, smoking, sedentary lifestyle, and term hypertension might lead to various cardiovascular diseases. The normal blood pressure, systolic, is 90-119 mm Hg and diastolic 60-79 mm Hg. The objective of this study is to find out the prevalence of hypertension among current cigarette smokers patients in an outpatient department of a tertiary care centre.

**Methods:**

A descriptive cross-sectional study was done on 385 outpatient department patients in a tertiary care centre from September, 2021 to February, 2022. The sample was collected by a convenience after approval from the Institutional Review Committee (Reference number: 0505202105). Outpatient department patients who had past history of smoking cigarette>100 cigarettes and who is still smoking were included in the study. Data were analysed by using the Statistical Package for the Social Science software version 16.0. Point estimate at 95% Confidence Interval was calculated along with frequency and percentage for binary data and mean with standard deviation for continuous data.

**Results:**

Among 385 patients, 209 (54.28%) (47.64-60.92 at 95% Confidence Interval) were hypertensive patients. One hundred fifty-six (40.51%) males and 53 (13.76%) females were hypertensive.

**Conclusions:**

The prevalence of hypertension was higher when compared to other studies done in similar settings.

## INTRODUCTION

Hypertension is a major public health issue in the world. Prolonged period of increase in blood pressure is hypertension. Normal systolic pressure is <120 mm of Hg, elevated is 120-129, stage 1 is 130-139, stage 2 is >140 and 180 is the stage of the crisis. The normal diastolic pressure is <80, stage 1 is 80-89, stage 2 is >90 and the stage of the crisis is 120.^1^ According to JNC 8 guidelines, ≥60 years patients without chronic disease, <150/90 mm Hg is normal. For patients 1859 years without comorbidities and ≥60 years with comorbidities, <140/90 mm Hg is normal.^2^

High blood pressure, smoking, and air pollution from household fuels are main risk factors for global disease burden.^3^ Hypertension is the major risk factor for cardiovascular disease.^4^ Smoking is the main factor for elevated blood pressure.^5^

The aim of this study is to find the prevalence of hypertension among current cigarette smokers in an outpatient department of a tertiary care centre.

## METHODS

A descriptive cross-sectional study was carried out among Out Patient Department (OPD) patients of Kathmandu Medical College, Duwakot, Bhaktapur. The study was started from September, 2021 to February, 2022 with the approval of the Institutional Review Committee (IRC) of Kathmandu Medical College and Teaching Hospital (Reference number: 0505202105). Patients who had past history of smoking >100 cigarettes and currently smoking were included in the study. Patients who are known case of hypertension and with normal blood pressure but under medication were considered hypertensive. The sample was collected by a convenience sampling. The sample size was calculated by using the following formula:

n = (Z^2^ × p × q) / e^2^

  = (1.96^2^ × 0.5 × 0.5) / 0.05^2^

  = 385

Where,

n = minimum required sample
Z = 1.96 for 95% Confidence Interval (CI)
p = prevalence taken as 50% for maximum
q = 1-p
e = margin of error, 5%

The minimum samples size of 385 was taken. Data were analysed by using the Statistical Package for the Social Science (SPSS) software version 16.0. Point estimate at 95% Confidence Interval was calculated along with frequency and percentage for binary data and mean with standard deviation for continuous data.

## RESULTS

**Figure 1:**
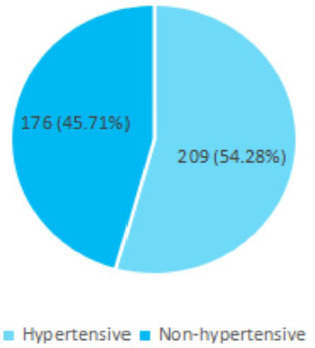
Frequency of hypertension among the total population (n= 385).

Out of 385 patients, 209 (54.28%) (47.64-60.92 at 95% Confidence Interval) were hypertensive patients and the mean age was 52.76±13.24 (Figure 1).

**Figure 2:**
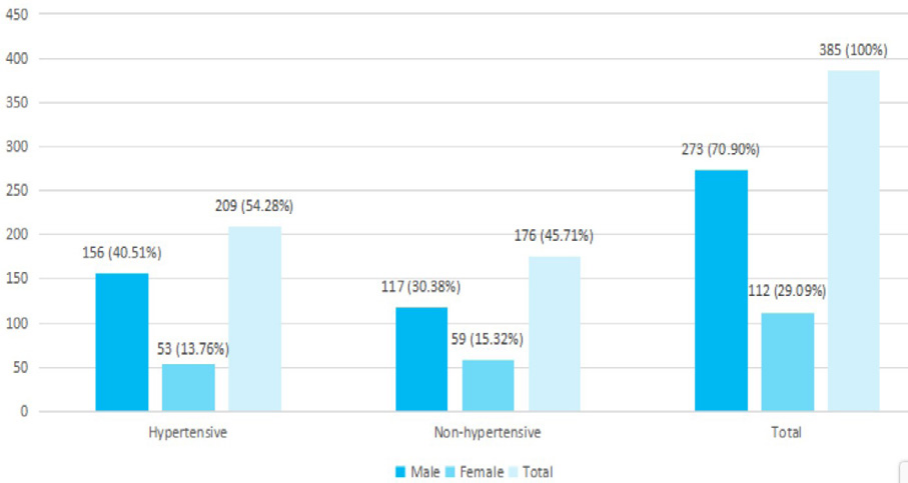
Frequency of hypertension among different genders (n= 385).

In total, hypertensive males were 156 (40.51%) and females were 53 (13.76%) respectively (Figure 2).

## DISCUSSION

In our findings, the prevalence of hypertension was 54.28% in the total sample. The prevalence was much higher in rate than in the Nepal Demographic and Health Survey (NDHS) 2016. NDHS showed no significant association with hypertension in smokers.^6^ The current study had a markedly higher frequency than a study that showed 19.8% in 1000 population.^7^ Similar findings were seen in a study done in a similar setting.^8^

In contrast to the current study, a study was done showed diastolic and mean arterial pressure in current smokers were lower than in non-smokers.^9^ An epidemiologic study showed higher blood pressure among nonsmokers and ex-smokers than current smokers.^10^ One of the studies showed about 38%,^11^ Two other studies showed significantly raised blood pressure in smokers.^12,13^ On the other hand, one of the studies showed modest relation of hypertension in smokers.^14^

This current study has some limitations, the sample size was relatively small and data was taken from only one tertiary care centre. The larger sample size in different sites might have greater values. The females and young subjects were hesitant to disclose their smoking habits due to social norms. So, there were higher chances of error in gender and age groups findings. In addition, different age groups, habits like exercise, alcohol intake, body mass index, and ethnicity might have greater and more significant values.

## CONCLUSIONS

The prevalence of hypertension among current cigarette smokers was higher when compared to other studies done in similar settings. Most of the males were hypertensive.
